# HP1a Targets the *Drosophila* KDM4A Demethylase to a Subset of Heterochromatic Genes to Regulate H3K36me3 Levels

**DOI:** 10.1371/journal.pone.0039758

**Published:** 2012-06-27

**Authors:** Chia-Hui Lin, Ariel Paulson, Susan M. Abmayr, Jerry L. Workman

**Affiliations:** 1 Stowers Institute for Medical Research, Kansas City, Missouri, United States of America; 2 Department of Anatomy and Cell Biology, University of Kansas Medical Center, Kansas City, Kansas, United States of America; Texas A&M University, United States of America

## Abstract

The KDM4 subfamily of JmjC domain-containing demethylases mediates demethylation of histone H3K36me3/me2 and H3K9me3/me2. Several studies have shown that human and yeast KDM4 proteins bind to specific gene promoters and regulate gene expression. However, the genome-wide distribution of KDM4 proteins and the mechanism of genomic-targeting remain elusive. We have previously identified *Drosophila* KDM4A (dKDM4A) as a histone H3K36me3 demethylase that directly interacts with HP1a. Here, we performed H3K36me3 ChIP-chip analysis in wild type and *dkdm4a* mutant embryos to identify genes regulated by dKDM4A demethylase activity in vivo. A subset of heterochromatic genes that show increased H3K36me3 levels in *dkdm4a* mutant embryos overlap with HP1a target genes. More importantly, binding to HP1a is required for dKDM4A-mediated H3K36me3 demethylation at a subset of heterochromatic genes. Collectively, these results show that HP1a functions to target the H3K36 demethylase dKDM4A to heterochromatic genes in *Drosophila*.

## Introduction

The post-translational modifications of histones play an important role in regulation of gene transcription and other cellular processes. Histone modifications not only affect the accessibility of histone-bound DNA, but also recruit individual proteins or protein complexes to discrete target sites in chromatin [1], [2]. Histone methylation at lysine residues is implicated in both gene activation and repression. The dynamic regulation of histone methylation by methyltransferases and demethylases, as well as their specificity toward different residues and methylation states (mono-, di-, or trimethylation), adds complexity to the function of histone methylation. Histone demethylases have been found to be involved in cellular differentiation and development, and are linked to several human diseases [3]. Thus, regulation of histone methylation is critical for cellular processes.

Histone H3K36 trimethylation (H3K36me3) is enriched in coding regions of actively transcribed genes [4], [5]. In *S. cerevisiae*, H3K36me3 facilitates histone deacetylation during transcription elongation, which in turn suppresses cryptic initiation within transcribed regions [6], [7], [8]. In *D. melanogaster*, H3K36me3 recruits the male-specific lethal (MSL) complex to dosage-compensated genes on the X chromosome in males [9], [10]. Recent studies in metazoans have shown that H3K36me3 is enriched on gene exons [11], [12], [13], [14]. In addition, H3K36me3 is implicated in regulation of alternative splicing [15]. Intriguingly, although H3K36me3 correlates with active transcription, this modification is also present in heterochromatic domains, where gene transcription is inactive, suggesting that it may contribute to the composition of heterochromatin [16].

The enzymes that catalyze demethylation of H3K36me3 belong to a large family of evolutionarily conserved Jumonji C (JmjC) domain-containing proteins [17]. The JmjC domain-containing proteins can be classified into subfamilies based on the alignment of JmjC domains. Generally, proteins within the same subfamily share the same residue specificity for histone demethylation [18]. The KDM4 subfamily mediates demethylation of histone H3K36me3/me2 and H3K9me3/me2 [19], [20], [21], [22]. It has recently been reported that KDM4 proteins also have demethylation activity on H1.4K26me3/me2 [23]. However, cellular functions and genomic targets of KDM4 proteins remain elusive.

KDM4 proteins are involved in regulation of gene expression and have been detected at some gene promoters. For example, human KDM4A binds to the ASCL2 gene promoter, where it functions as an N-CoR-associated corepressor [24]. In addition, human KDM4B functions as a co-regulator for estrogen receptor (ER) signaling [25]. The yeast KDM4 ortholog, Rph1, regulates H3K36 methylation at actively transcribed regions, where it plays a positive role in transcription elongation [26]. A recent study showed that Rph1 associates with the PHR1 gene promoter through its zinc finger domains to regulate the level of H3K36me3, resulting in repression of PHR1 expression [27]. Despite these studies on individual genes, little is known about the genome-wide distribution of KDM4 proteins and the mechanism of targeting to their target genes. While domains within human or yeast KDM4 might function in targeting KDM4 to chromatin [27], [28], [29], *Drosophila* KDM4A and KDM4B lack the PHD, Tudor and zinc finger domains that are found in other KDM4 orthologs [18], [30]. Thus, mechanisms of targeting KDM4 to the genome might differ between *Drosophila* KDM4 and its orthologs.

We previously demonstrated that *Drosophila* KDM4A is a functional histone H3K36me3/me2 demethylase that directly interacts with Heterochromatin Protein 1a (HP1a) through a consensus HP1 binding motif, PxVxL, at its C terminus [31]. HP1a was first identified in *D. melanogaster* as a non-histone chromosome binding protein that predominantly localizes to the chromocenter on polytene chromosomes [32]. HP1a functions as a dominant suppressor of position effect variegation (PEV), suggesting it plays a role in the formation and spread of heterochromatin [33]. While HP1a has been well characterized as a “repressive” mark of gene transcription [34], [35], it also functions in gene activation at both heterochromatic and euchromatic loci [36], [37], [38], [39]. Notably, both cytological and genomic data support a role for HP1a in regulating euchromatic loci [40], [41], [42], [43]. Our previous finding that dKDM4A interacts with HP1a suggested that HP1a might target dKDM4A to chromatin to demethylate H3K36me3. Herein we performed H3K36me3 ChIP-chip analysis in wild type and *dkdm4a* mutant embryos to identify candidate genes regulated by dKDM4A demethylase activity in vivo. By comparing H3K36me3 ChIP-chip analysis with known HP1a binding sites [44], we identified a subset of genes regulated by both dKDM4A and HP1a. Using a mutant form of dKDM4A defective for HP1a-binding, we further demonstrate that dKDM4A regulates H3K36me3 levels through binding to HP1a at a subset of heterochromatic genes.

## Results

### Identification of dKDM4A Target Genes by H3K36me3 ChIP-chip Analysis

**Figure 1:**
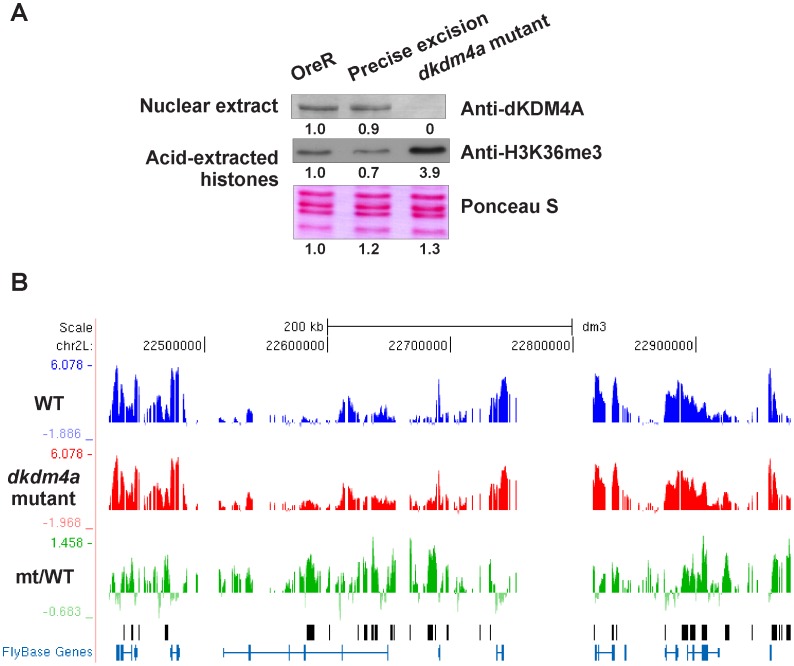
Identification of dKDM4A targets by H3K36me3 ChIP-chip analysis. (A) P-element insertion abrogates the expression of dKDM4A and results in increased levels of bulk histone H3K36me3. Precise excision of P-element restores the expression level of dKDM4A and the bulk level of H3K36me3. Nuclear extracts and acid-extracted histones from embryos of OreR, *dkdm4a* mutant and precise excision rescued fly lines were analyzed by western blot using indicated antibodies. Quantitation of the signal intensity is shown below each blot. (B) H3K36me3 profiles of chromosome 2 Lh genes. H3K36me3 ChIP-chip results are displayed on the UCSC genome browser for one representative biological replicate. The profile of H3K36me3 ChIP-chip in wild type embryos is shown in blue and the profile in *dkdm4a* mutant embryos is shown in red. The profile of increased H3K36me3 levels (mt/WT) is shown in green. Peaks called on the ratio track (mt/WT) are marked with black bars. y-axis shows log2 intensity ratio values; x-axis shows protein-coding genes annotated by Flybase.

To identify dKDM4A target genes in vivo, we examined genome-wide changes in H3K36me3 levels in wild type and *dkdm4a* mutant embryos. The *dkdm4a* allele contains a P-element inserted within the first exon of the gene and abrogates *dKDM4A* transcription [31], [45]. We were also unable to detect dKDM4A protein in *dkdm4a* mutant embryos (Figure 1A), suggesting that this mutation represents a null allele. As anticipated, this loss of dKDM4A correlates with increased levels of bulk histone H3K36me3 in embryos (Figure 1A). To avoid variation in genetic background between control and mutant embryos, we generated a fly line with a precise excision of the original P-element insertion, thereby recreating an intact dKDM4A gene in a chromosomal background that is identical to that of the mutant. This excision line was used as the control (wild type) in our ChIP-chip analysis. Excision of the P transposon completely restores the expression level of dKDM4A and the bulk level of H3K36me3 (Figure 1A).

To examine genome-wide changes in H3K36me3, we performed chromatin immunoprecipitation using an antibody against H3K36me3 and chromatin isolated from early *dkdm4a* mutant and P-element excision (wild type) embryos (2–4 hr after egg laying), followed by microarray analysis (ChIP-chip). Immunoprecipitated DNA was labeled and hybridized along with input DNA on high-density genomic tiling microarrays. Reproducibility of two biological replicates is shown in Figure S1A. The H3K36me3 peaks in wild type embryos significantly overlap with previously identified H3K36me3 profile peaks in 2–4 hr embryos of the Oregon R strain [44] (Figure S1B). An example of H3K36me3 profiles of chromosome 2 L observed in wild type and *dkdm4a* mutant embryos is shown in Figure 1B. We identified 834 positive peaks at which the H3K36me3 level is increased in the absence of dKDM4A relative to wild type. We verified more than 20 peak regions from the top ranked peaks by ChIP-qPCR, and they all showed increased H3K36me3 levels in the *dkdm4a* mutant (data not shown). These 834 peaks correspond to 658 genes, which represent putative genes regulated by dKDM4A demethylase activity in 2–4 hr embryos.

### dKDM4A and HP1a Target Heterochromatic Genes

Previous studies have shown that dKDM4A directly interacts with HP1a, and that this association stimulates dKDM4A H3K36me3 demethylation activity [31]. Thus, we next asked whether dKDM4A and HP1a target the same genes in vivo. To identify genes regulated by both dKDM4A and HP1a, we compared peaks indicating increased H3K36me3 levels in *dkdm4a* mutant embryos with previously identified HP1a binding sites [44]. The HP1a ChIP-chip was performed using chromatin from 2–4 hr wild-type embryos of the Oregon R strain. The overlapping peaks between the two datasets were extracted. If multiple neighboring peaks of HP1a binding overlapped with a single H3K36me3 peak, we combined the HP1a binding sites into one peak, and vice versa. This analysis revealed 145 peaks of HP1a enrichment in wild type embryos and increased levels of H3K36me3 in *dkdm4a* mutant embryos. These 145 peaks correspond to 69 candidate target genes co-regulated by HP1a and dKDM4A.

**Figure 2:**
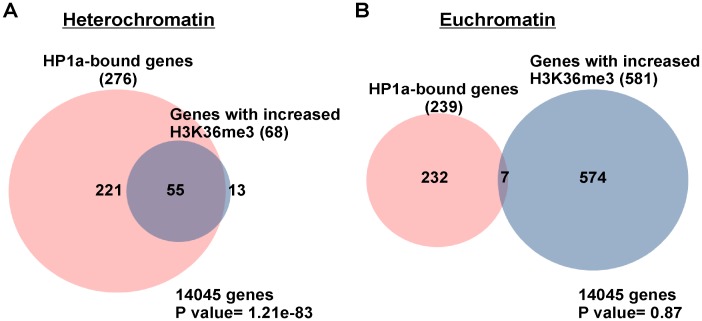
Identification of Common Target Genes of dKDM4A and HP1a. The Venn diagram analysis of heterochromatic genes (A) or euchromatic genes (B) bound by HP1a in wild type and genes with increased H3K36me3 levels in *dkdm4a* mutant embryos. Genes assigned to the chromosome U are not included in the analysis. The P value was calculated by the hypergeometric test.

**Figure 3:**
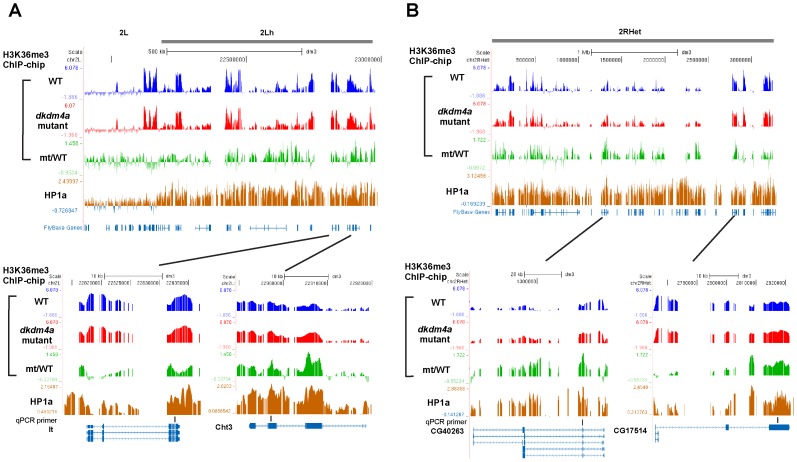
Profiles of H3K36me3 and HP1a ChIP-chip at heterochromatin. H3K36me3 and HP1a profiles of chromosome 2 Lh (A) and 2 RHet (B) genes. Profiles of H3K36me3 ChIP-chip are shown as in Figure 1B. The profile of HP1a ChIP-chip is shown in brown. Primers used in Figure 4B are indicated in the panel of qPCR primers.

To examine whether dKDM4A functions with HP1a at heterochromatin or euchromatin, we applied the definition of heterochromatin based on Release 5 of the *D. melanogaster* genome sequence [46], [47] and epigenomic euchromatin-heterochromatin borders [48]. There are three classes of heterochromatic sequences defined in Release 5 of the *D. melanogaster* genome: (1) sequences assembled contiguously with the euchromatic arms (“h”; e.g., 2 Lh), (2) scaffolds mapped to a specific chromosome arm with partial information on order and orientation (“Het”; e.g., 2 LHet), and (3) unmapped sequences (arm U) [46], [47]. In addition to cytological criteria, sharp transitions of H3K9me2 defined by ChIP-chip analysis were used to determine epigenomic euchromatin-heterochromatin borders [48]. According to the above definitions, we found that among the 69 common target genes, 55 genes reside within heterochromatic domains (“h” and “Het”, and chromosome 4), and 7 genes are within chromosome U, while there are 7 euchromatic genes. Since chromosome U contains highly repetitive and unmapped sequences, genes assigned to chromosome U were excluded in the following analysis. We used Venn diagram analysis to compare the list of genes with either increased H3K36me3 levels in *dkdm4a* mutant embryos or bound by HP1a at heterochromatin or euchromatin. We observed an overlap between the two gene lists for the heterochromatin genes (P value = 1.21e-83), suggesting that HP1a functions in targeting dKDM4A activity to heterochromatin (Figure 2A). In contrast, the demethylation activity of dKDM4A at euchromatin is independent of HP1a targeting, since the two gene lists at euchromatin is not highly correlated (P value = 0.87) (Figure 2B). Profiles of H3K36me3 and HP1a ChIP-chip of chromosome 2 Lh and 2 RHet, as well as profiles at genes *light* (*lt)*, *Chitinase 3* (*Cht3)*, *CG40263* and *CG17514*, are shown in Figure 3. The ChIP-chip profile shows that HP1a-enriched heterochromatic domains are enriched for H3K36me3, and peaks representing increased H3K36me3 levels in *dkdm4a* mutant embryos (mt/WT, the green track in Figure 3) are observed at these heterochromatic domains. Thus, dKDM4A and HP1a commonly target a subset of heterochromatic genes.

### Binding to HP1a is Required for dKDM4A-mediated H3K36me3 Demethylation at a Subset of Heterochromatic Genes

**Figure 4:**
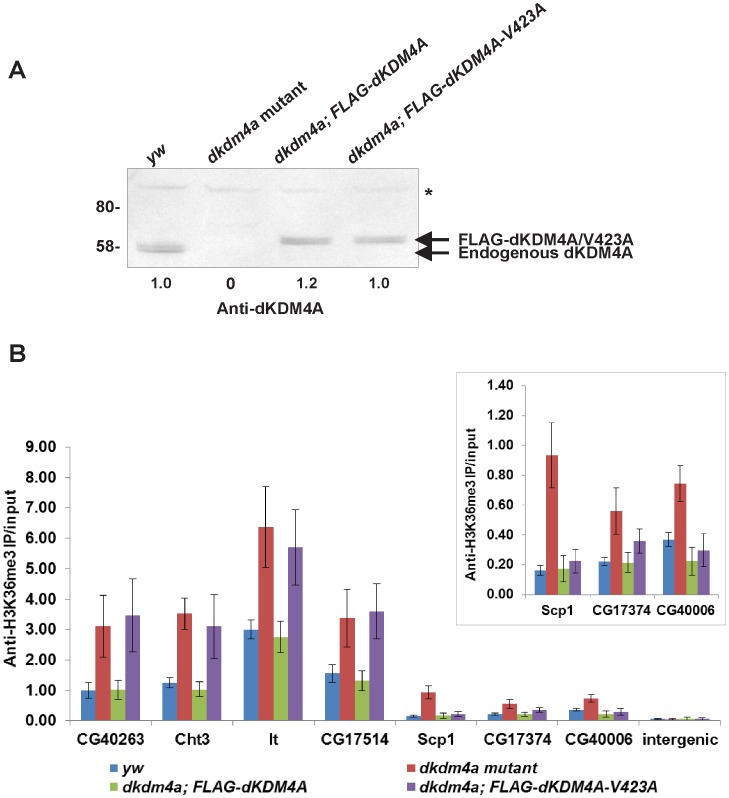
dKDM4A-mediated H3K36me3 demethylation requires HP1a binding at a subset of heterochromatic genes. (A) The expression level of endogenous dKDM4A and FLAG-tagged dKDM4A or dKDM4A-V423A. Nuclear extracts from embryos of *yw*, *dkdm4a* mutants and mutants rescued by FLAG-dKDM4A were analyzed by western blot. The doublet band is likely to represent two isoforms of dKDM4A. Asterisk indicates non-specific signal. Quantitation of the signal intensity is shown below the blot. (B) The enrichment of H3K36me3 at HP1a-bound heterochromatic genes was observed by ChIP-qPCR. Genes with lower H3K36me3 levels (*Scp1, CG17374* and *CG40006*) are also shown in a separate panel. The primer set amplifying an intergenic region at chromosome 2 L was used as a negative control. The error bars represent standard deviation from 3 biological repeats.

To confirm the regulation of H3K36me3 levels by dKDM4A at HP1a-bound heterochromatic genes, we examined the enrichment of H3K36me3 at candidate common target genes of dKDM4A and HP1a in *dkdm4a* mutant embryos, and in mutants rescued by expression of FLAG-tagged dKDM4A. The *yw67c23* (*yw*) fly line was used as a control in the ChIP assay since it is the parental line of the P-element insertion mutant. In the genomic rescue fly line, FLAG-tagged dKDM4A is expressed at endogenous levels (Figure 4A). We examined the enrichment of H3K36me3 by ChIP-qPCR at gene regions that are among the top ranked peaks of increased H3K36me3 in *dkdm4a* mutants (Figure 4B). As expected, the level of H3K36me3 increased at HP1a-bound heterochromatic genes in *dkdm4a* mutant embryos. The increased H3K36me3 levels were rescued by expressing FLAG-dKDM4A in *dkdm4a* mutant embryos (Figure 4B), suggesting that dKDM4A regulates the level of H3K36me3 at these HP1a-bound heterochromatic genes.

**Figure 5:**
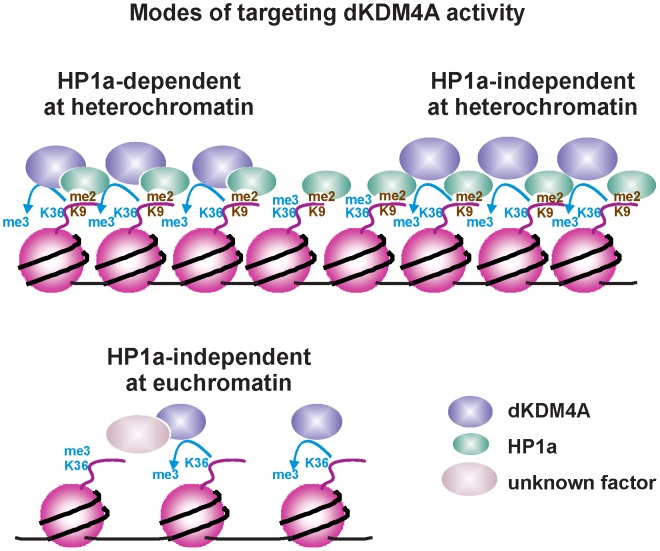
Model for HP1a targeting dKDM4A activity to a subset of heterochromatic genes. Demethylation of H3K36me3 by dKDM4A requires binding to HP1a at a subset of heterochromatic loci. Other mechanisms might exist to target dKDM4A activity to euchromatin, as well as heterochromatin. For example, dKDM4A might be targeted to euchromatin through other protein factors.

To determine whether the interaction between HP1a and dKDM4A is required for targeting dKDM4A activity to these heterochromatic genes, we next examined H3K36me3 levels at heterochromatic genes in mutants with the genomic rescue transgene that contains a point mutation (V423A). This point mutation at the central valine of PxVxL motif disrupts the interaction between dKDM4A and HP1a in vitro [31]. The expression level of the FLAG-tagged dKDM4A-V423A is comparable to that observed in *yw* and FLAG-dKDM4A genomic rescue (Figure 4A). When we examined the enrichment of H3K36me3 by ChIP-qPCR, it showed that the HP1a-binding mutant form of dKDM4A failed to rescue the increased level of H3K36me3 at some of the heterochromatic genes (Figure 4B, *CG40263*, *Cht3*, *lt* and *CG17514*). We also observed rescue of H3K36me3 levels that is independent of dKDM4A binding to HP1a at *Sarcoplasmic calcium-binding protein 1* (*Scp1)*, *CG17374* and *CG40006*, where the level of H3K36me3 is lower than that at *CG40263*, *Cht3*, *lt* and *CG17514* (Figure 4B). Thus, based on our results, binding to HP1a is required for demethylation of H3K36me3 by dKDM4A at a subset of heterochromatic genes. However, unknown mechanisms exist to target dKDM4A activity to euchromatic targets and also to some heterochromatic domains (Figure 5).

## Discussion

In this study, we examined the H3K36me3 levels genome-wide in wild type and *dkdm4a* mutant embryos to identify candidate target loci regulated by dKDM4A, a histone H3K36me3/me2 demethylase that associates with HP1a. A subset of heterochromatic genes that show increased H3K36me3 levels in *dkdm4a* mutant embryos overlap with HP1a target genes. While expression of dKDM4A in these embryos rescued H3K36me3 levels at heterochromatic genes, expression of a mutant form of dKDM4A which is unable to bind to HP1a (dKDM4A-V423A) failed to rescue H3K36me3 levels at some of the heterochromatic genes. Thus, binding to HP1a is required for dKDM4A to catalyze demethylation of H3K36me3 at a subset of heterochromatic genes.

While candidate genes regulated by dKDM4A demethylase activity were identified using H3K36me3 ChIP-chip analysis, we were unable to detect dKDM4A recruitment directly using anti-dKDM4A antibodies in the ChIP assay (data not shown). Since the inability of our anti-dKDM4A antibodies to work efficiently in ChIP assays could account for this result, we also performed ChIP assays using anti-FLAG antibody with chromatin from FLAG-dKDM4A genomic rescue embryos. However, again, we were unable to detect enrichment of FLAG-dKDM4A at genes with increased levels of H3K36me3 in *dkdm4a* mutant embryos (data not shown). We speculate that the binding of dKDM4A to its target genomic loci might be transient and undetectable in ChIP assays because of the rapid nature of the enzymatic reactions. In fact, in a previous study of the yeast KDM4 homolog, Rph1, no enrichment of Rph1 was detected by ChIP analysis, despite increased H3K36me3 levels across the gene body of actively transcribed genes were observed in *rph1*Δ mutants [26].

Although we observe increased levels of H3K36me3 across the genome in the absence of dKDM4A, *dkdm4a* mutant flies are homozygous viable and fertile. We speculate that dKDM4B, the other *Drosophila* KDM4 ortholog, is able to compensate for the absence of dKDM4A in these mutant flies. dKDM4B exhibits demethylation activity on both H3K36me3/me2 and H3K9me3/me2 [31], [49]. A similar scenario has been reported for *kdm4d*-null mice [50]. Loss of KDM4D, a testis-enriched H3K9me3 demethylase, results in accumulation of H3K9me3 in round spermatids and dramatic changes in distribution of methylated H3K9 in germ cells. However, no fertility defects were observed in *kdm4d*-null mice. Since there are four KDM4 proteins in mammals, functional redundancy might rescue possible defects in *kdm4d*-null mice [50].

Based on our data, we propose that dKDM4A mediates H3K36me3 demethylation at a subset of heterochromatic genes through binding to HP1a. Interestingly, the level of H3K36me3 at these gene targets remains highly enriched in wild type embryos. One would expect to see H3K36me3 significantly reduced, if not depleted, at dKDM4A target genes, since dKDM4A functions as an H3K36me3 demethylase and its activity can be stimulated by HP1a binding. The pattern of H3K36me3 we observe in H3K36me3 ChIP-chip analysis suggests that dKDM4A demethylation activity might function to fine-tune H3K36me3 levels at heterochromatic loci. By performing H3K36me3 ChIP followed by real-time PCR at heterochromatic genes with chromatin from wild type, *dkdm4a* mutant and genomic rescued embryos, we found that while expressing wild-type dDKM4A in the mutant can rescue increased H3K36me3 levels, dKDM4A binding to HP1a is only required for H3K36me3 demethylation at genes with higher wild-type levels of H3K36me3 (*CG40263*, *Cht3*, *lt*, and *CG17514*). Genes with low wild-type levels of H3K36me3 (*Scp1*, *CG17374*, *CG40006*) seem to be regulated by dKDM4A independent of HP1a binding (Figure 4B). This observation suggests that different mechanisms for targeting dKDM4A to substrates might apply within heterochromatic domains, as well as at euchromatin. Alternatively, while the stimulation of dKDM4A demethylation activity by HP1a is required at genes that are highly enriched for H3K36me3, the intrinsic demethylation activity of dKDM4A might be sufficient to regulate the level of H3K36me3 at genes with low H3K36me3 enrichment.

In our H3K36me3 ChIP-chip analysis, genes with increased levels of H3K36me3 in the absence of dKDM4A are likely targeted by dKDM4A and regulated by its demethylation activity in vivo. However, based on our RNA-seq analysis, dKDM4A-regulated heterochromatic genes do not show changes in gene expression in *dkdm4a* mutant embryos, and only 23 euchromatic dKDM4A targets show changes in gene expression more than two fold in *dkdm4a* mutant embryos (data not shown). Moreover, several heterochromatic loci targeted by both HP1a and dKDM4A are within intergenic regions (Figure S2), suggesting that heterochromatic regulation of H3K36me3 levels may contribute to the structure of the heterochromatin or cellular processes other than gene transcription. In addition to its involvement in transcriptional regulation and establishment of heterochromatic structure, HP1a also functions in regulation of DNA replication and DNA repair at heterochromatin [51]. A genome-wide study of the role of HP1a in modulating replication timing showed that knockdown of HP1a results in delayed replication timing at HP1a target regions, including the 4th chromosome and pericentric regions [52]. Thus, regulation of H3K36me3 levels by HP1a and dKDM4A might contribute to modulation of replication timing at heterochromatic loci. In addition, HP1a is involved in DNA repair of double-strand breaks (DSBs) at heterochromatin [53], [54]. DSBs that occurred in heterochromatin are repaired by homologous recombination [55]. Notably, heterochromatic DSBs relocalize to outside of heterochromatin to complete DNA repair mediated by Rad51, preventing recombination among repetitive sequences within heterochromatin. HP1a is required to recruit Smc5/6 complex to heterochromatin to prevent the formation of Rad51 foci [55]. Therefore, it is possible that the regulation of H3K36me3 levels by HP1a and dKDM4A contributes to the DNA repair process at heterochromatin.

In conclusion, this study demonstrates an in vivo function for the interaction between dKDM4A and HP1a, which is to target dKDM4A demethylation activity to HP1a-bound heterochromatic loci. Although H3K36me3 correlates with active transcription in general, regulation of this modification in heterochromatin by HP1a and dKDM4A might involve in other cellular processes.

## Materials and Methods

### Fly Stocks and Crosses

The *dkdm4a* mutant fly stock (*y^1^ w^67c23^; P{y^+mDint2^ w^BR.E.BR^ = SUPor-P}Kdm4A^KG04636^*) was obtained from the Bloomington Stock Center at Indiana University (stock number 13828) [56], [57]. The P-element KG04636 was mobilized by crossing to y^1^w^*^; CyO, H{w^[+mc]^ = PΔ2–3}Hop2.1/Bc^1^Egfr^E1^. P-element excision was screened by loss of the eye color marker associated with the transgene, and further confirmed by PCR. PCR products were sequenced to confirm precise excision.

To generate the dKDM4A genomic rescue, a fragment containing the genomic dKDM4A locus including about 1.6 kilobases upstream of 5′ UTR and 220 bp downstream of 3′ UTR of dKDM4A was amplified from the genomic DNA of Oregon R flies. Double FLAG tags were added at the C-terminus of dKDM4A. The V423A mutation was generated using Quick Change II XL Site-Directed Mutagenesis Kit (Stratagene). The fragment was cloned into the pCa4B vector [58]. Site specific integration at the attP40 landing site (2 L 25C7) [58] was carried out by Genetic Services. To rescue the *dkdm4a* mutant, the second chromosome transgene (FLAG-dKDM4A or dKDM4A-V423A) was recombined with the KG04636 insertion.

### Generation of Antibodies to dKDM4A

The anti-dKDM4A antibody was generated by immunizing rabbits and guinea pigs with the synthetic peptide CVPEPSSAPKRYDFNTEAVVRV conjugated with KLH (keyhole limpet hemocyanin). (Pocono Rabbit Farm and Laboratory Inc.).

### Preparation of Nuclear Extracts and Histones from *Drosophila* Embryos

6–18 hr embryos were dechorionated in 50% bleach, homogenized in Buffer I (15 mM Hepes pH 7.5, 10 mM KCl, 5 mM MgCl_2_, 0.1 mM EDTA, 0.5 mM EGTA, 350 mM sucrose, and protease inhibitors), and filtered through a single layer of miracloth prior to centrifugation 10,400×g 15 min 4°C. The soluble nuclear fraction was isolated by resuspending nuclei in Extraction Buffer (20 mM Hepes pH 7.5, 10% glycerol, 350 mM NaCl, 1 mM MgCl2, 0.1% TritonX-100 and protease inhibitors) for 1 hr 4°C with rotation, followed by centrifugation to pellet the insoluble chromatin fraction at 14,000 rpm 10 min 4°C. Acid-soluble material was extracted from the insoluble chromatin fraction by resuspending the pellet in 0.4 M HCl, followed by centrifugation. The supernatant containing the histone proteins was neutralized by adding an equimolar volume of NaOH.

### ChIP

ChIP was performed from staged 2–4 hr embryos collected in population cages as described in [59], [60] with minor modifications. Briefly, embryos were cross linked in 1.8% formaldehyde and homogenized in Buffer A1 (15 mM Hepes pH 7.5, 15 mM NaCl, 60 mM, KCl, 4 mM MgCl, 0.5% Triton X-100, 0.5 mM DTT and protease inhibitors). Nuclei were resuspended in A2 buffer (15 mM Hepes pH 7.5, 140 mM NaCl, 1 mM EDTA, 0.5 mM EGTA, 1% Triton X-100, 0.1% sodium deoxycholate, 1% SDS, 0.5% N-lauroylsarcosine and protease inhibitors) and sonicated to obtain chromatin fragments with an average size of ∼500 bp. For input controls, 50 µl of chromatin was used. About 700 µg to 1 mg of chromatin was incubated with 1.5 µg anti-H3K36me3 antibody (abcam, ab9050) overnight at 4°C. 30 µl of sheep anti-rabbit IgG Dynabeads was added to each chromatin/antibody solution and incubated for 2 hr 4°C, then beads were washed 5 times with RIPA Buffer (50 mM Hepes pH 7.5, 0.5 M LiCl, 1 mM EDTA, 1% NP-40, 0.7% sodium deoxycholate) and once with 50 mM NaCl in TE. Bound complexes were eluted twice with 200 µl of elution buffer (50 mM Tris pH 8.0, 10 mM EDTA, 1% SDS) at 65°C for 30 min. The eluates were treated with RNase A (0.2 µg/µl) for 1 hr at 37°C followed by Proteinase K treatment (0.2 µg/µl) for 1 hr at 55°C. Crosslinks were reversed by incubating samples at 65°C overnight. DNA was purified by phenol-chloroform extraction and ethanol precipitation. Input and immunoprecipitated (IP) DNA samples were analyzed by real-time PCR. The primer sequences are listed in Table S1.

### ChIP-chip Analysis

Two biological replicates of H3K36me3 ChIPs were performed in *dkdm4a* mutant and wild type (P-element revertant) embryos. The amplification and labeling of immunoprecipitated DNA and input DNA were performed as described in [61]. Cy5-labeled IP DNA and Cy3-labeled input DNA were hybridized to *Drosophila* whole genome ChIP-on-chip microarrays (Agilent) using Agilent CGH protocol and reagents. Two slides of 244 K microarrays containing probes tiled across whole *Drosophila* genome with 233 nt average spacing. Peaks were called on the ratio track (mt/WT) using a double-threshold method. Track was smoothed using a 5-probe MA, then the mean and SD for positive-valued probes were calculated. Peaks were called using a candidate threshold of 1 SD outside the mean and a peak threshold of 2 SD outside the mean, with a minimum run of 3 probes, max gap = 1000 bp. In other words, any contiguous run of more than 3 probes, with heights at or beyond 1 SD above the mean, and having no internal gaps >1000 bp, becomes a candidate. Any candidate with at least one probe at or beyond 2 SD is called a peak. To find positive peaks (increased H3K36me3 levels in *dkdm4a* mutant embryos) which are consistently present in both replicates, only peaks that are positive, overlapping a peak in the other replicate, and contain more positive probes than negative in the mutant data are retained. Peaks were matched to genes with at least 1 bp overlap. ChIP-chip data are MIAME compliant. Raw data has been deposited in a MIAME compliant database accessible through NCBI’s Gene Expression Omnibus [62] (GSE37016). HP1a and H3K36me3 ChIP-chip in wild type embryos of Oregon R strain were obtained from modMine (DCCid: modENCODE 2665 and 932).

## Supporting Information

Figure S1 **Reproducibility of H3K36me3 ChIP-chip.** (A) Scatter plots showing correlation between probe values (log2 IP/input) of replicate 1 versus replicate 2 for wild type (WT) and mutant profiles. The plots show strong correlation between two biological replicates. (B) The Venn diagram analysis of peaks called on wild type track of H3K36me3 ChIP-chip and peaks called using same criteria on H3K36me3 profile of 2–4 hr embryos of the Oregon R strain from modENCODE project.
(TIF)

Figure S2 **The distribution of heterochromatic loci targeted by both HP1a and dKDM4A.** The diagram illustrating the overall distribution of peaks of HP1a enrichment in wild type embryos and increased levels of H3K36me3 in *dkdm4a* mutant embryos at heterochromatin. Peaks overlap a gene more than 50% are in the category of “gene body”, while peaks overlap a gene less than 50% are in the category of “gene-overlapping.” TSS, transcription start site; TES, transcription end site.
(TIF)

Table S1 **Primers used in ChIP-qPCR.**
(DOCX)
