# Supplementary figures and images for: HP1a Targets the Drosophila KDM4A Demethylase to a Subset of Heterochromatic Genes to Regulate H3K36me3 Levels
Source: PLoS One. 2012 Jun 27;7(6):e39758. doi: 10.1371/journal.pone.0039758 (PMC3384587; doi:10.1371/journal.pone.0039758)

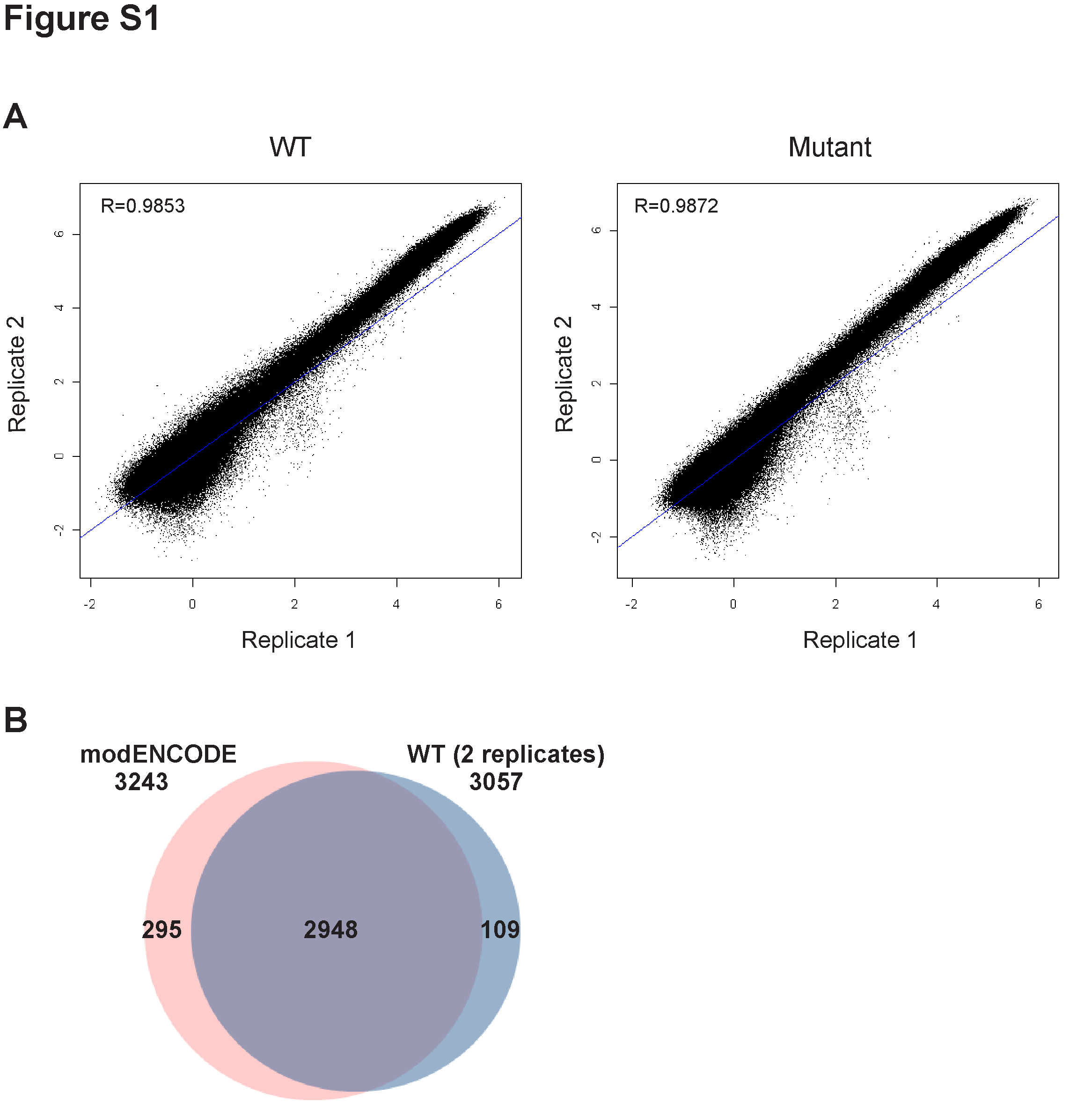

Supplement: Figure S1 — Reproducibility of H3K36me3 ChIP-chip. (A) Scatter plots showing correlation between probe values (log2 IP/input) of replicate 1 versus replicate 2 for wild type (WT) and mutant profiles. The plots show strong correlation between two biological replicates. (B) The Venn diagram analysis of peaks called on wild type track of H3K36me3 ChIP-chip and peaks called using same criteria on H3K36me3 profile of 2–4 hr embryos of the Oregon R strain from modENCODE project. (TIF) [file pone.0039758.s001.tif]

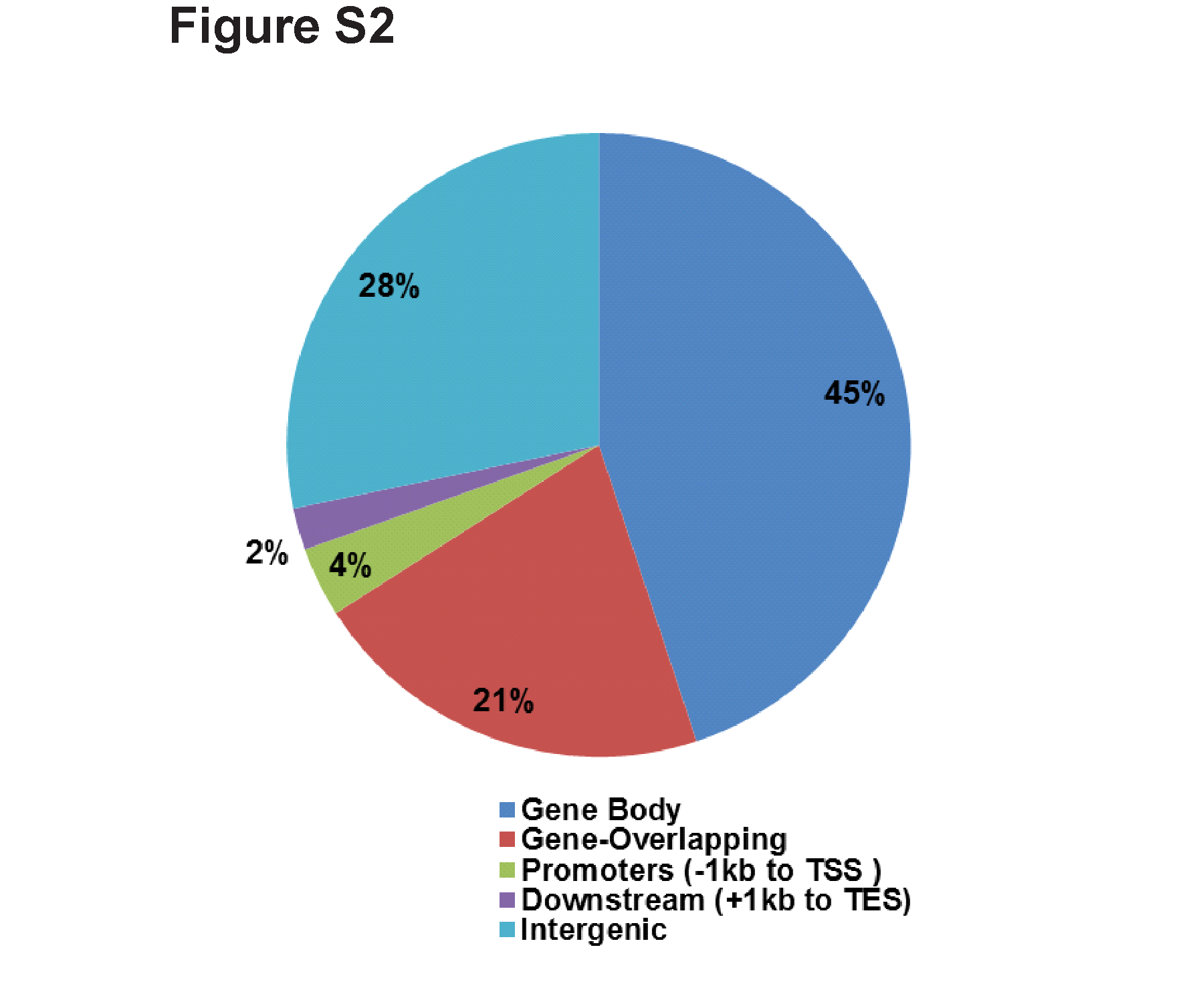

Supplement: Figure S2 — The distribution of heterochromatic loci targeted by both HP1a and dKDM4A. The diagram illustrating the overall distribution of peaks of HP1a enrichment in wild type embryos and increased levels of H3K36me3 in dkdm4a mutant embryos at heterochromatin. Peaks overlap a gene more than 50% are in the category of “gene body”, while peaks overlap a gene less than 50% are in the category of “gene-overlapping.” TSS, transcription start site; TES, transcription end site. (TIF) [file pone.0039758.s002.tif]
