# Supplementary material for: HP1a Targets the Drosophila KDM4A Demethylase to a Subset of Heterochromatic Genes to Regulate H3K36me3 Levels
Source: PLoS One. 2012 Jun 27;7(6):e39758. doi: 10.1371/journal.pone.0039758 (PMC3384587; doi:10.1371/journal.pone.0039758)
Supplement: Table S1 — Primers used in ChIP-qPCR. (DOCX) [file pone.0039758.s003.docx]

**Table S1. Primers used in ChIP-qPCR**

| Primer Name | FlyBase accession number | | Sequence |
| --- | --- | --- | --- |
| Scp1.1 | FBgn0020908 | | GCAGAACCCATCAGCAAAAT |
| Scp1.2 |  |  | AGGGAATTGCTTTTCCGAGT |
| CG40263.1 | FBgn0058263 | | TACCAACGCCCTGAATTCTC |
| CG40263.2 |  |  | CCTCGCTTCTTCGTGGTAAG |
| Cht3.1 | FBgn0250907 | | CGTGGGAGCATTTAGTTGGT |
| Cht3.2 |  |  | ATGTACAGCCACCCAGAAGG |
| CG40006.1 | FBgn0058006 | | CTGTACTGCCCTTGTGCTGA |
| CG40006.2 |  |  | TGTGACTGAAGCGGCTAATG |
| lt.1 | FBgn0002566 | | GGTCACAATTTCAGGGTGCT |
| lt.2 |  |  | AGAAGCGTCCGAAGCACTTA |
| CG17374.1 | FBgn0040001 | | GGTGGTCTGACTGCTCAACA |
| CG17374.2 |  |  | GCCATTATGACAAGCCGTTT |
| CG17514.1 | FBgn0039959 | | TCAGACTTCGCCAATGAGTG |
| CG17514.2 |  |  | AATGGGTTCGTTAGCACGTC |
| intergenic.1 |  |  | AATTGCATCGCAACACAATGAG |
| intergenic.2 |  |  | TCGTGAAATGTTTGCTACTGGAATA |
